# Mandibular Clinical Arch Forms in Iraqi Population: A National Survey

**DOI:** 10.3390/diagnostics12102352

**Published:** 2022-09-28

**Authors:** Munther A. Ali, Yassir A. Yassir

**Affiliations:** 1 Department of Orthodontics, College of Dentistry, University of Baghdad, Baghdad 10047, Iraq; 2 Orthodontic Department, College of Dentistry, University of Baghdad, Baghdad 10047, Iraq; 3 School of Dentistry, University of Dundee, Dundee DD1 4HN, UK

**Keywords:** arch dimensions, arch measurements, Iraqi population

## Abstract

**Background:** This study aims to identify mandibular clinical arch forms and dimensions in the Iraqi population. **Materials and methods:** The study sample consisted of pre-treatment mandibular study models of the Iraqi population. The most labial aspect of 13 proximal contact areas was digitized using AutoCAD software to determine the clinical bracket point for every tooth. The dental arches were classified into three types: tapered, ovoid, and square. The arch dimensions were identified using four linear and two proportional measurements. **Results:** A total of 1005 study models were collected. The arch forms were distributed as ovoid (47%), tapered (36.2%), and square (16.8%), with no significant difference in the distribution between Arabs and Kurds. The ovoid arch form was predominant in class I and class III malocclusion, while the tapered arch form was predominant in class II. All the linear measurements were greater in the males than in the females. The arch widths decreased as the arch form shifted from square to ovoid to tapered, while the arch depths showed the reverse relation. **Conclusions:** According to this study, ovoid and tapered archwires should suit the majority of Iraqi patients. The ovoid arch form was the most predominant in the subjects with class I and class III malocclusion, while the tapered arch form was the most predominant in the class II subjects.

## 1. Introduction

One of the most common causes of relapse after orthodontic treatment is the alteration of the patient’s original arch form [1,2]. The tendency for post-treatment relapse will be higher if greater changes in the arch form occur during treatment [1]. The preservation of the original intercanine and intermolar width is critical for stability since it represents the position of the teeth that results from the muscular balance of each patient [3]. When the same archwire form is used for all patients, the initial arch form is likely to change, resulting in an unstable result [2]. Moreover, preformed superelastic archwires are difficult to adapt to each patient’s specific arch form [4]; so, having a variety of preformed archwires on hand is more practical [5]. It is crucial to identify and choose the shape that most resembles the patient’s pre-treatment arch form, according to the race or type of malocclusion, during the alignment and leveling stage [6] to attain stable, functional, and esthetic outcomes [7].

The determination of arch forms from the pre-treatment mandibular arch has been common [4,6,8,9,10,11,12,13] as there are more therapeutic limitations in the mandibular dental arch than in the maxillary arch, and maintaining the mandibular intercanine width is essential for stable orthodontic treatments [14,15]; thus, investigating arch dimensions becomes necessary [16]. Many authors have described the arch form using geometric shapes and mathematical formulas [17,18]. Others used landmarks such as incisal edges and cusp tips to characterize an individual patient’s arch form [16,19,20,21,22,23]. These approaches may not be adequately precise because archwires are placed on the slot point of the brackets, which are located on the facial axis points. Therefore, recent research has used clinical bracket points for arch form analysis, which seems more clinically relevant [4,6,9,11,12,13].

The various ethnic and racial groups are subjected to different environmental factors and exhibit different genetic and developmental features, and this may be reflected as a variation in the size and shape of dental arches [24]. Additionally, arch form differences between ethnic groups have also been found in several studies [4,6,9,12,13,25]. Therefore, the accurate arch form determination has become of special importance between various racial groups.

As no study to date has been conducted to identify the dental arch form for the Iraqi population, this study aimed to determine the mandibular clinical arch forms in the Iraqi population according to the following objectives:

1. Identifying the most common mandibular arch form in Iraqis (via a national survey) and comparing between the main ethnic groups (Arab and Kurdish).
2. Comparing arch forms and dimensions according to gender, age, and type of malocclusion.

The null hypothesis stated that “there is no significant difference in the arch forms of Arab and Kurdish Iraqis.”

## 2. Materials and Methods

The sample of this study consisted of pre-treatment mandibular study models of Iraqi populations from the main ethnic groups (Arab and Kurd). This was categorized according to gender and type of malocclusion (Angle’s class I, class II, and class III), with the age being ≥13 years old. The sample was collected from teaching hospitals in addition to different private orthodontic clinics from 11 Iraqi governorates according to the criteria below:

### 2.1. Inclusion Criteria

1. Class I, II, and III malocclusions (Angle’s classification).
2. Entire permanent dentition, except for the third molars.
3. No prominent teeth malformations.
4. No local factors affecting the integrity of the dental arches (e.g., supernumerary teeth, retained deciduous teeth, and congenitally missing teeth).
5. Arch length discrepancy of ≤3 mm.
6. No previous orthodontic treatment, orthognathic surgery, or fixed prosthodontic therapy.
7. Adequate quality of study models with no deformation, fractures, or air bubbles.

### 2.2. Exclusion Criteria

1. The presence of prosthetic replacement or restoration extensions to cusp tips/incisal edges or cervical areas.
2. History of facial/dental trauma.
3. Severe transverse arch discrepancies.
4. Patients with cleft palate.
5. Moderate and severe crowding or spacing.

**Figure 1:**
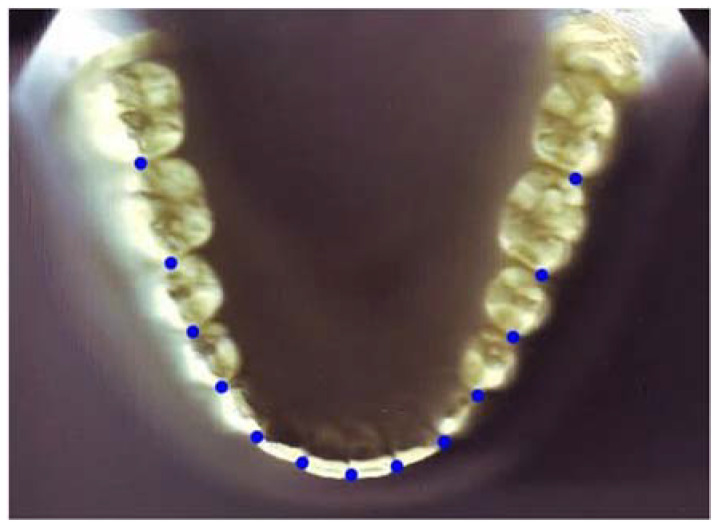
Digitized contact points on a mandibular cast.

The selected models were scanned digitally using the Canon Canoscan Lide25 scanner (1200 dpi) with a ruler fixed for magnification correction. The most labial aspect of the 13 proximal contact areas within the arch was digitized with the AutoCAD computer software (Autodesk^®^ 2020) (Figure 1). The X- and Y-coordinates were generated from the point of contact between the two central incisors. Adjustments of the original X- and Y- axes were performed so that line A (a horizontal line that joins the left and right contact points between the first and second premolars) and line B (a horizontal line that joins the left and right contact points between the second premolars and the first molars) were parallel to the *X*-axis.

**Figure 2:**
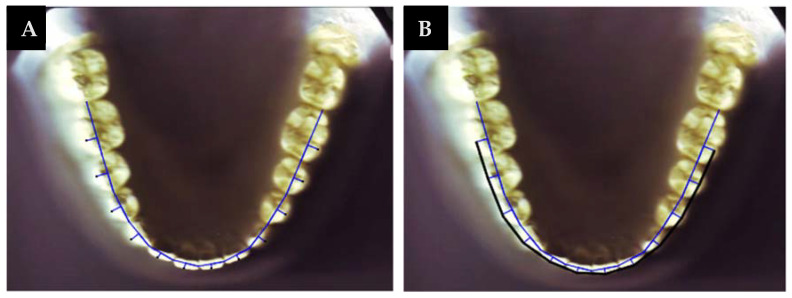
(**A**) Clinical bracket points; (**B**) contact point and clinical bracket point lines.

**Figure 3:**
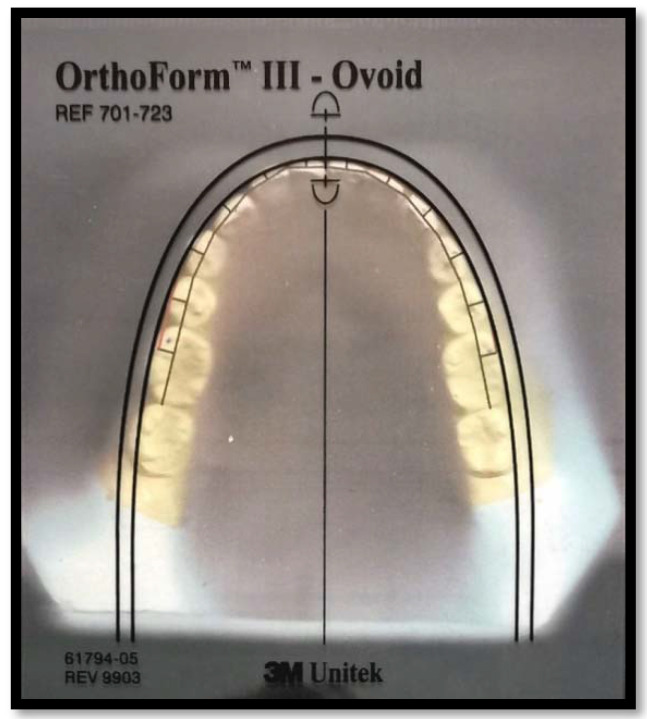
Superimposition of clear orthoform template on a printed digital model.

To establish the clinical bracket points for every anterior tooth and premolar, a perpendicular line was drawn to extend facially from the midpoint of the line that connects the mesial and distal contact points [4,6,9,12]. This was based on Andrew’s data on the prominence of the crown [26]. In the molars, this line was drawn from the region where the mesial third meets the distal two-thirds. Another line connecting the clinical bracket points was drawn to determine the arch form (Figure 2). Afterwards, the digital model was printed on a 1:1 scale. Then, the three different arch forms (tapered, ovoid, and square) were identified using the 3M Unitek templates. This was carried out relying on an arch form that provides a proper fit for the eight clinical bracket points from the right to the left first premolars [27] (Figure 3).

The evaluated proportional and linear measurements were:

1. *Intercanine width:* The distance between the clinical bracket points of the canines.
2. *Intermolar width:* The distance between the clinical bracket points of the first molars.
3. *Canine depth:* The smallest distance from a point between the central incisors to a line joining the clinical bracket point of the canines.
4. *Molar depth:* The smallest distance from a point between the central incisors to a line joining the clinical bracket point of the first molars.
5. *Canine W/D ratio:* This represents the intercanine width to the canine depth ratio.
6. *Molar W/D ratio:* This represents the intermolar width to the molar depth ratio.

### 2.3. Statistical Analysis

The Statistical Package for Social Sciences for Windows, version 25.0 (SPSS Inc., Chicago, IL, USA), was used for the statistical analysis.

Mean, standard deviation, frequency, and percentages were used to describe the data. The inter- and intra-examiner reliability of the digital measurement compared to the direct measurement was tested using an intraclass correlation coefficient (ICC) for 10 study models (tested twice with a four-week interval), while the inter- and intra-examiner reliability of the arch form determination was performed using the weighted kappa test for 10 study models (tested twice with a four-week interval). The following inferential statistics were used:

- *Chi-square test:* To compare arch forms among ethnic groups, genders, and types of malocclusion and to compare genders between the ethnic groups and the malocclusions between ethnic groups and gender.
- *Independent samples t-test*: To compare age and arch dimensions between ethnic groups and gender.
- *Analysis of variance (ANOVA) and Tukey post hoc tests*: To compare arch dimensions among different arch forms and Angle classifications.

The level of significance was set as *p* < 0.05.

## 3. Results

A total of 1005 study models were collected (452 class I, 391 class II, and 196 class III). The total sample comprised 659 females and 346 males, with a mean age of 18 years old. The Arab group consisted of 822 subjects, and the Kurd group had 183 subjects.

The frequency and percentage of the sample distribution are shown in Table 1. Class I malocclusion had the highest frequency distribution, followed by class II and class III, respectively. Regarding the arch form distribution in the total sample, the ovoid arch form was the most frequently seen (47%), followed by the tapered (36.2%) then the square (16.8%). Both ethnic groups followed the same pattern of arch form distribution with no statistically significant difference between them (*p* = 0.331). The ovoid arch form was the most frequently seen in both genders; a square arch form was the least frequently seen in the females, while the tapered and square arch forms were evenly distributed in the males (*p* = 0.000). In terms of malocclusion, the ovoid arch form was the most frequently seen in class I and class III, followed by the tapered then the square in class I and the square then the tapered in class III, while the tapered arch form was the most frequently seen in class II, followed by the ovoid then the square (*p* = 0.000) (Table 2).

There were no statistically significant differences in gender distribution between the two ethnic groups (*p* = 0.169); both ethnic groups had more females than males, representing 66.5% of the Arab group and 61.2% of the Kurd group (Table 3). Similarly, there was no statistically significant difference in malocclusion distribution between the two ethnic groups (*p* = 0.058), while, the malocclusion distribution between the genders significantly differed (*p* = 0.002) (Table 4).

The Arab group had a significantly higher molar depth (*p* = 0.044) and a smaller molar W/D ratio (*p* = 0.021) compared to the Kurd group, while there were no statistically significant differences in the other arch dimensions and in age between the two ethnic groups (Table 5). Age and all the linear measurements were greater in the males than in the females (Table 6). However, this was not statistically significant for the age and canine W/D ratio.

The results showed statistically significant differences in arch dimensions among the three arch forms (Table 7 and Table 8), while age was almost similar among them. Intercanine width, intermolar width, canine W/D ratio, and molar W/D decreased as the mandibular arch form shifted from square to ovoid to tapered, while canine depth and molar depth increased as the mandibular arch form shifted from square to ovoid to tapered.

The subjects with class I malocclusion were statistically significantly older than the class II subjects (*p* = 0.000). The intercanine width in class III was greater than the class II malocclusion (*p* = 0.004), while the intermolar width in class III was greater than in class I (*p* = 0.003) and class II (*p* = 0.000). The canine depth was greater in class II than in class I (*p* = 0.005) and class III (*p* = 0.000). On the other hand, the canine W/D ratio and molar W/D ratios were higher in class III than in the class I and II malocclusions. Additionally, the canine W/D ratio was higher in class I than in class II (Table 9 and Table 10).

## 4. Discussion

The availability of preformed archwires with three predominant shapes (tapered, ovoid, and square) necessitates the use of a suitable archwire that follows the pre-treatment arch form of the patients to obtain a stable, functional, and esthetic result, unless the expansion is required for the constricted arch.

Some researchers investigated the Iraqi population’s arch dimensions and arch form [22,28,29,30]. The current study has been conducted as no previous study in the literature had performed a national survey to determine the pre-treatment mandibular arch forms for the Iraqi population.

As the findings of this study did not reach the statistical significance between the ethnic groups in terms of arch form, there was insufficient evidence to reject the null hypothesis.

Regarding sample size and distribution, the total sample size was considered large compared to the other studies with similar aims [4,6,7,9,11,12,13,16,20,21,23,25,31]. The females represented 65.6% of the total sample. This higher percentage of females in comparison to males may reflect the higher percentage in the population and the greater interest of females in having a better facial and dental appearance. Gender distribution was almost similar between both ethnic groups.

In this investigation, a 2D scanner was used, and this method was also used in other studies [2,4,20,32,33,34] as it is simple and practical for the large sample size. Similarly, arch form templates were used in evaluating the mandibular arch forms as they have been commonly used in orthodontic clinics to choose orthodontic prefabricated archwires for patients [8].

The ovoid arch form was the most frequently seen, followed by the tapered and then the square in both ethnic groups. The ovoid arch form was also the most frequently seen in both genders. However, the results found significant differences between the males and females in the distribution of other arch forms. The square arch form was the least frequently seen in females, while the tapered and square arch forms were evenly distributed in males. The higher percentage of square arch form in males than females may be attributed to the larger arch dimensions in males than females.

The ovoid arch form was the most predominant in class I and class III, followed by the tapered then the square in class I and the square then the tapered in class III, while the tapered arch form was the most predominant in class II, followed by the ovoid then the square. These results are consistent with other studies in that there was an increased frequency of the tapered arch form and a decreased frequency of the ovoid arch form in class II compared to the class I arches [4,6,9,12,13,25]. This may be due to the compensation of the dentition in the class II malocclusion to decrease the increased overjet by proclination of the lower anterior teeth [35,36], making the arch closer to the tapered form. However, there is disagreement with other studies that noted small differences among the arch forms of the class I and class II groups [11]. There was a general tendency to find more square arch forms in the class III group compared to the class I and II group, which was consistent with the findings of other studies [4,6,9,11,12,13,25].

Class III malocclusion usually occurs due to mandibular prognathism, in which the mandibular dimensions are increased. This could explain the above finding since larger arch dimensions characterize the square arch form compared to the tapered and ovoid arch forms. Furthermore, Nojima et al. [9] supposed that there is a “common pathogenesis of class III malocclusion and the resultant dental compensation by lingual tipping of the mandibular anterior teeth, causing the anterior part of the mandibular arch to flatten.”

It has been shown that there were no statistically significant differences in age and all arch dimensions between the two ethnic groups except in the molar depth and molar W/D ratio (the Arab group had greater molar depth). As a consequence of the greater molar depth, Arabs have a smaller molar W/D ratio compared to the Kurd group. Nevertheless, the two ethnic groups showed an almost identical canine W/D ratio, indicating that their dental arches have similar anterior curvature.

All the linear measurements were greater in the males than the females. This finding could explain the higher percentage of the square arch form in males and the tapered arch form in females, and it agreed with Olmez and Dogan [11], who found that arch width and depth were greater in boys than in girls. According to Younes [37], this may be due to girls having a smaller and smoother bone ridge and alveolar processes, as well as their average weaker musculature, which all play a role in face breadth measurements, dental arch width, and height.

Intercanine width, intermolar width, canine W/D ratio, and molar W/D decreased as the mandibular arch form shifted from square to ovoid to tapered, while canine depth and molar depth increased as the mandibular arch form shifted from square to ovoid to tapered. These results seem logical as arch width increased when arch form increased in width (tapered-to-square), and the opposite was the case for arch depth. As the number of teeth is the same, a wider arch is usually associated with shallower depth and vice versa; this is supported by Gafni et al. [12], who found that the widest and shallowest canine and molar dimensions were found in the square arch form, whereas the narrowest and deepest canine and molar dimensions were found in the tapered arch form.

The class I subjects were significantly older than the class II subjects. Shaw [38] found that the degree of apparent occlusal irregularity was the most important factor of satisfaction with dental appearance and the desire for orthodontic treatment. As class II malocclusion is usually associated with increased overjet, this may dissatisfy the patients and/or their parents and enhance the motivation for early orthodontic treatment.

There was no statistically significant difference among the Angles classes regarding molar depth. This was in agreement with Bayomi et al. [4], who also could not find such a significant difference. Class III arches were found to have a wider intercanine width than class II arches. Likewise, the intermolar width in class III was greater than in classes I and II. This can be explained by lingual tipping of the anterior teeth and flattening of the anterior region in class III development, as well as lateral tongue growth owing to decreased arch depth [6,11,39]. Class II subjects had significantly larger canine depth than class I and III subjects; this might be due to a slightly tapered anterior curvature that influences canine depth directly. It was also shown that the canine W/D ratio of class II arches is the least, followed by class I and class III. In addition, the greatest molar W/D ratio is seen in the class III arches, followed by the class I and II arches. These findings can be attributed to the relation between arch form and arch width and depth, as the class II subjects predominantly had tapered arch form; these narrower arches are usually associated with a smaller width and greater depth, while the class III subjects have a higher percentage of the square arch form, which is characterized by the greater width and shallower depth. This is consistent with the results by Gafni et al. [12].

### Strengths and Limitations of the Study

This study can be considered the first study that has been conducted as a national survey of the pre-treatment mandibular arch forms in the Iraqi population and compared between Arab and Kurd. It included a large sample size of 1005 subjects from 11 Iraqi governorates.

This study also has some limitations, such as the use of a 2D method to assess arch form. Despite being clinically appropriate, it could be improved with the use of a 3D method of assessment. Moreover, the sample of this study included orthodontic patients only.

This study concluded with the following:

1. Ovoid was the most frequent arch form (in this Iraqi sample), followed by the tapered then the square arch forms.
2. There was no statistically significant difference regarding arch form distribution between the Arab and Kurd subjects.
3. The ovoid arch form was the most predominant in the subjects with class I and class III malocclusion, followed by the tapered then the square in class I and the square then the tapered in the class III subjects, while the tapered arch form was the most predominant in the class II subjects, followed by the ovoid and then the square arch forms.
4. Males, generally, have larger dental arch measurements than females.
5. The subjects with class III malocclusion had the greatest intercanine and intermolar widths and the lowest canine and molar depths. Canine depth was greater in the subjects with class II malocclusion than in those with class I and III malocclusions.
6. The arch form should be carefully considered when selecting archwire during orthodontic treatment according to the current results and the type of malocclusion; so, having archwires with three forms (ovoid, tapered, and square) is necessary in order to choose the most appropriate archwire that is identical with the patient pre-treatment arch form. This in turn could increase the stability of the treatment and minimize the chance of relapse.

## Figures and Tables

**Table 1:** Frequency and percentages of sample distribution.

Variable		Number	Percent
**Governorate**	**Baghdad**	420	41.8%
	**Dayala**	25	2.5%
	**Babylon**	20	2.0%
	**Karbala**	21	2.1%
	**Maisan**	20	2.0%
	**Nasiriyah**	61	6.1%
	**Basrah**	141	14.0%
	**Naynawa**	73	7.3%
	**Kirkuk**	41	4.1%
	**Erbil**	64	6.4%
	**Duhuk**	119	11.8%
Iraqi Regions	**North of Iraq**	297	29.6%
	**Middle of Iraq**	487	48.5%
	**South of Iraq**	221	22.0%
Race	**Arab**	822	81.8%
	**Kurd**	183	18.2%
Gender	**Female**	659	65.6%
	**Male**	346	34.4%
Malocclusion	**Class I**	452	45.0%
	**Class II**	391	38.9%
	**Class III**	162	16.1%
Arch Form	**Ovoid**	472	47.0%
	**Tapered**	364	36.2%
	**Square**	169	16.8%
Total		**1005**	**100.0%**

**Table 2:** Descriptive statistics and comparison of arch form distribution (chi-square test).

			Arch Form			Total	*p*-Value
			Ovoid	Tapered	Square		
**Race**	**Arab**	**Count**	**393 _a_**	289 _a_	140 _a_	822	0.331
		**Expected Count**	386.1	297.7	138.2	822.0	
		**% within Race**	47.8%	35.2%	17.0%	100.0%	
	**Kurd**	**Count**	79 _a_	75 _a_	29 _a_	183	
		**Expected Count**	85.9	66.3	30.8	183.0	
		**% within Race**	43.2%	41.0%	15.8%	100.0%	
Gender	**Female**	**Count**	302 _a_	276 _b_	81 _c_	659	0.000 ***
		**Expected Count**	309.5	238.7	110.8	659.0	
		**% within Gender**	45.8%	41.9%	12.3%	100.0%	
	**Male**	**Count**	170 _a_	88 _b_	88 _c_	346	
		**Expected Count**	162.5	125.3	58.2	346.0	
		**% within Gender**	49.1%	25.4%	25.4%	100.0%	
Malocclusion	**Class I**	**Count**	233 _a_	146 _b_	73 _a,b_	452	0.000 ***
		**Expected Count**	212.3	163.7	76	452	
		**% within Malocclusion**	51.5%	32.3%	16.2%	100.0%	
	**Class II**	**Count**	158 _a_	184 _b_	49 _a_	391	
		**Expected Count**	183.6	141.6	65.8	391	
		**% within Malocclusion**	40.4%	47.1%	12.5%	100.0%	
	**Class III**	**Count**	81 _a_	34 _b_	47 _c_	162	
		**Expected Count**	76.1	58.7	27.2	162	
		**% within Malocclusion**	50.0%	21.0%	29.0%	100.0%	

Each subscript letter denotes a subset of Arch form categories whose column proportions do not differ significantly from each other at the 0.05 level. *** (Very highly significant).

**Table 3:** Descriptive statistics and comparison of gender distribution between races (chi-square test).

			Gender		Total	*p*-Value
			Female	Male		
Race	**Arab**	**Count**	547 _a_	275 _a_	822	0.169
		**Expected Count**	539.0	283.0	822.0	
		**% within Race**	66.5%	33.5%	100.0%	
	**Kurd**	**Count**	112 _a_	71 _a_	183	
		**Expected Count**	120.0	63.0	183.0	
		**% within Race**	61.2%	38.8%	100.0%	

Each subscript letter denotes a subset of Gender categories whose column proportions do not differ significantly from each other at the 0.05 level.

**Table 4:** Descriptive statistics and comparison of malocclusions distribution (chi-square test).

			Malocclusion			Total	*p*-Value
			Class I	Class II	Class III		
Race	**Arab**	**Count**	381 _a_	318 _a_	123 _a_	822	0.058
		**Expected Count**	369.7	319.8	132.5	822.0	
		**% within Race**	46.4%	38.7%	15.0%	100.0%	
	**Kurd**	**Count**	71 _a_	73 _a_	39 _a_	183	
		**Expected Count**	82.3	71.2	29.5	183.0	
		**% within Race**	38.8%	39.9%	21.3%	100.0%	
Gender	**Female**	**Count**	291 _a,b_	278 _b_	90 _a_	659	0.002 **
		**Expected Count**	296.4	256.4	106.2	659.0	
		**% within Gender**	44.2%	42.2%	13.7%	100.0%	
	**Male**	**Count**	161 _a,b_	113 _b_	72 _a_	346	
		**Expected Count**	155.6	134.6	55.8	346.0	
		**% within Gender**	46.5%	32.7%	20.8%	100.0%	

Each subscript letter denotes a subset of Malocclusion categories whose column proportions do not differ significantly from each other at the 0.05 level. ** (Highly significant).

**Table 5:** Descriptive statistics and comparison of age and arch dimensions between races (independent samples *t*-test).

Variables	Race	N	Mean	SD	SE	t	*p*-Value	Mean Difference	95% CI	
									Lower	Upper
Age	**Arab**	822	17.89	3.77	0.13	−1.767	0.078	−0.58	−1.23	0.07
	**Kurd**	183	18.48	4.08	0.30					
Intercanine Width	**Arab**	822	29.48	1.61	0.06	1.346	0.179	0.18	−0.08	0.44
	**Kurd**	183	29.30	1.63	0.12					
Intermolar Width	**Arab**	822	48.18	2.80	0.10	−0.734	0.463	−0.16	−0.60	0.27
	**Kurd**	183	48.35	2.43	0.18					
Canine Depth	**Arab**	822	5.88	1.08	0.04	−0.184	0.854	−0.02	−0.19	0.16
	**Kurd**	183	5.90	1.13	0.08					
Molar Depth	**Arab**	822	26.31	2.19	0.08	2.017	0.044 *	0.36	0.01	0.70
	**Kurd**	183	25.95	2.02	0.15					
Canine W/D Ratio	**Arab**	822	5.18	1.01	0.04	0.022	0.982	0.00	−0.17	0.17
	**Kurd**	183	5.18	1.27	0.09					
Molar W/D Ratio	**Arab**	822	1.84	0.16	0.01	−2.307	0.021 *	−0.03	−0.06	0.00
	**Kurd**	183	1.87	0.15	0.01					

* (Significant).

**Table 6:** Descriptive statistics and comparison of age and arch dimensions between genders (independent samples *t*-test).

Variables	Gender	N	Mean	SD	SE	t	*p*-Value	Mean Difference	95% CI	
									Lower	Upper
Age	**Female**	659	17.90	3.91	0.15	−1.144	0.253	−0.29	−0.79	0.21
	**Male**	346	18.19	3.69	0.20					
Intercanine Width	**Female**	659	29.16	1.51	0.06	−8.018	0.000 ***	−0.83	−1.04	−0.63
	**Male**	346	29.99	1.66	0.09					
Intermolar Width	**Female**	659	47.51	2.55	0.10	−12.060	0.000 ***	−2.05	−2.38	−1.71
	**Male**	346	49.55	2.57	0.14					
Canine Depth	**Female**	659	5.82	1.04	0.04	−2.259	0.024 *	−0.17	−0.32	−0.02
	**Male**	346	5.99	1.17	0.06					
Molar Depth	**Female**	659	26.02	2.18	0.09	−4.627	0.000 ***	−0.66	−0.94	−0.38
	**Male**	346	26.67	2.05	0.11					
Canine W/D Ratio	**Female**	659	5.18	1.05	0.04	−0.321	0.748	−0.02	−0.16	0.12
	**Male**	346	5.20	1.08	0.06					
Molar W/D Ratio	**Female**	659	1.84	0.16	0.01	−2.919	0.004 **	−0.03	−0.05	−0.01
	**Male**	346	1.87	0.16	0.01					

* (Significant), ** (Highly significant), *** (Very highly significant).

**Table 7:** Descriptive statistics and comparison of age and arch dimensions among arch forms (ANOVA test).

Variables	Arch Form	N	Mean	SD	SE	Min	Max	F	*p*-Value
Age	**Ovoid**	472	18.07	3.64	0.168	13	30	0.188	0.829
	**Tapered**	364	17.96	4.12	0.216	13	32		
	**Square**	169	17.88	3.72	0.286	13	29		
	**Total**	1005	18.00	3.83	0.121	13	32		
Intercanine Width	**Ovoid**	472	29.67	1.42	0.07	26.16	33.78	105.399	0.000 ***
	**Tapered**	364	28.65	1.41	0.07	23.41	32.68		
	**Square**	169	30.53	1.70	0.13	26.70	35.24		
	**Total**	1005	29.44	1.61	0.05	23.41	35.24		
Intermolar Width	**Ovoid**	472	48.56	2.47	0.11	26.12	55.44	195.273	0.000 ***
	**Tapered**	364	46.59	2.09	0.11	41.42	53.56		
	**Square**	169	50.75	2.37	0.18	44.74	58.50		
	**Total**	1005	48.21	2.73	0.09	26.12	58.50		
Canine Depth	**Ovoid**	472	5.71	0.89	0.04	3.23	8.55	200.852	0.000 ***
	**Tapered**	364	6.56	0.93	0.05	3.73	9.38		
	**Square**	169	4.91	1.00	0.08	2.12	7.87		
	**Total**	1005	5.88	1.09	0.03	2.12	9.38		
Molar Depth	**Ovoid**	472	26.12	2.26	0.10	20.96	52.64	32.171	0.000 ***
	**Tapered**	364	26.83	1.82	0.10	19.94	33.95		
	**Square**	169	25.30	2.18	0.17	18.94	33.39		
	**Total**	1005	26.24	2.16	0.07	18.94	52.64		
Canine W/D Ratio	**Ovoid**	472	5.30	0.71	0.03	3.68	9.11	376.787	0.000 ***
	**Tapered**	364	4.44	0.54	0.03	2.66	6.55		
	**Square**	169	6.46	1.34	0.10	4.41	14.68		
	**Total**	1005	5.18	1.06	0.03	2.66	14.68		
Molar W/D Ratio	**Ovoid**	472	1.87	0.14	0.01	0.89	2.30	264.716	0.000 ***
	**Tapered**	364	1.74	0.11	0.01	1.44	2.14		
	**Square**	169	2.02	0.15	0.01	1.57	2.48		
	**Total**	1005	1.85	0.16	0.01	0.89	2.48		

*** (Very highly significant).

**Table 8:** Post hoc Tukey HSD test showing the comparison of arch dimensions between two of each arch form.

Variables	Arch Form		Mean Difference	SE	*p*-Value	95% CI	
						Lower	Upper
Intercanine Width	**Ovoid**	**Tapered**	1.02	0.10	0.000 ***	0.78	1.26
	**Ovoid**	**Square**	−0.87	0.13	0.000 ***	−1.17	−0.56
	**Tapered**	**Square**	−1.88	0.14	0.000 ***	−2.20	−1.56
Intermolar Width	**Ovoid**	**Tapered**	1.97	0.16	0.000 ***	1.59	2.35
	**Ovoid**	**Square**	−2.19	0.21	0.000 ***	−2.68	−1.70
	**Tapered**	**Square**	−4.16	0.22	0.000 ***	−4.67	−3.66
Canine Depth	**Ovoid**	**Tapered**	−0.86	0.06	0.000 ***	−1.01	−0.71
	**Ovoid**	**Square**	0.79	0.08	0.000 ***	0.60	0.99
	**Tapered**	**Square**	1.65	0.09	0.000 ***	1.45	1.85
Molar Depth	**Ovoid**	**Tapered**	−0.71	0.15	0.000 ***	−1.05	−0.36
	**Ovoid**	**Square**	0.82	0.19	0.000 ***	0.38	1.26
	**Tapered**	**Square**	1.53	0.20	0.000 ***	1.07	1.99
Canine W/D Ratio	**Ovoid**	**Tapered**	0.87	0.06	0.000 ***	0.73	1.00
	**Ovoid**	**Square**	−1.16	0.07	0.000 ***	−1.32	−0.99
	**Tapered**	**Square**	−2.02	0.07	0.000 ***	−2.20	−1.85
Molar W/D Ratio	**Ovoid**	**Tapered**	0.13	0.01	0.000 ***	0.10	0.15
	**Ovoid**	**Square**	−0.15	0.01	0.000 ***	−0.18	−0.12
	**Tapered**	**Square**	−0.27	0.01	0.000 ***	−0.30	−0.25

*** (Very highly significant).

**Table 9:** Descriptive statistics and comparison of age and arch dimensions among malocclusions (ANOVA test).

Variables	Malocclusion	N	Mean	SD	SE	Min	Max	F	*p*-Value
Age	**Class I**	452	18.55	3.87	0.18	13	32	9.996	0.000 ***
	**Class II**	391	17.38	3.64	0.18	13	30		
	**Class III**	162	17.95	3.97	0.31	13	30		
	**Total**	1005	18.00	3.83	0.12	13	32		
Intercanine Width	**Class I**	452	29.51	1.59	0.07	24.71	35.24	5.785	0.003 **
	**Class II**	391	29.25	1.57	0.08	23.41	34.22		
	**Class III**	162	29.73	1.74	0.14	26.16	34.87		
	**Total**	1005	29.44	1.61	0.05	23.41	35.24		
Intermolar Width	**Class I**	452	48.23	2.87	0.13	26.12	56.92	11.562	0.000 ***
	**Class II**	391	47.84	2.58	0.13	41.42	58.50		
	**Class III**	162	49.05	2.55	0.20	43.78	55.85		
	**Total**	1005	48.21	2.73	0.09	26.12	58.50		
Canine Depth	**Class I**	452	5.83	1.02	0.05	3.23	9.38	11.354	0.000 ***
	**Class II**	391	6.06	1.16	0.06	2.12	9.14		
	**Class III**	162	5.61	1.03	0.08	3.07	8.33		
	**Total**	1005	5.88	1.09	0.03	2.12	9.38		
Molar Depth	**Class I**	452	26.32	2.28	0.11	21.68	52.64	0.719	0.487
	**Class II**	391	26.22	2.00	0.10	18.94	32.18		
	**Class III**	162	26.08	2.17	0.17	20.41	33.39		
	**Total**	1005	26.24	2.16	0.07	18.94	52.64		
Canine W/D Ratio	**Class I**	452	5.21	0.94	0.04	3.24	9.25	11.046	0.000 ***
	**Class II**	391	5.03	1.16	0.06	2.66	14.68		
	**Class III**	162	5.48	1.04	0.08	3.81	9.54		
	**Total**	1005	5.18	1.06	0.03	2.66	14.68		
Molar W/D Ratio	**Class I**	452	1.84	0.16	0.01	0.89	2.48	7.593	0.001 **
	**Class II**	391	1.83	0.16	0.01	1.45	2.42		
	**Class III**	162	1.89	0.16	0.01	1.57	2.33		
	**Total**	1005	1.85	0.16	0.01	0.89	2.48		

** (Highly significant), *** (Very highly significant).

**Table 10:** Post hoc Tukey HSD test showing the comparison of age and arch dimensions between malocclusions.

Variables	Malocclusion		Mean Difference	SE	*p*-Value	95% CI	
						Lower	Upper
Age	**Class I**	**Class II**	1.17	0.26	0.000 ***	0.56	1.79
	**Class I**	**Class III**	0.60	0.35	0.194	−0.21	1.42
	**Class II**	**Class III**	−0.57	0.35	0.244	−1.40	0.26
Intercanine Width	**Class I**	**Class II**	0.26	0.11	0.051	0.00	0.52
	**Class I**	**Class III**	−0.22	0.15	0.292	−0.57	0.12
	**Class II**	**Class III**	−0.48	0.15	0.004 **	−0.83	−0.13
Intermolar Width	**Class I**	**Class II**	0.39	0.19	0.091	−0.05	0.83
	**Class I**	**Class III**	−0.82	0.25	0.003 **	−1.40	−0.24
	**Class II**	**Class III**	−1.21	0.25	0.000 **	−1.81	−0.62
Canine Depth	**Class I**	**Class II**	−0.24	0.07	0.005 **	−0.41	−0.06
	**Class I**	**Class III**	0.22	0.10	0.066	−0.01	0.45
	**Class II**	**Class III**	0.46	0.10	0.000 ***	0.22	0.69
Canine W/D Ratio	**Class I**	**Class II**	0.19	0.07	0.026 *	0.02	0.36
	**Class I**	**Class III**	−0.27	0.10	0.016 *	−0.49	−0.04
	**Class II**	**Class III**	−0.45	0.10	0.000 ***	−0.68	−0.22
Molar W/D Ratio	**Class I**	**Class II**	0.01	0.01	0.712	−0.02	0.03
	**Class I**	**Class III**	−0.05	0.01	0.003 **	−0.08	−0.01
	**Class II**	**Class III**	−0.06	0.02	0.000 ***	−0.09	−0.02

* (Significant), ** (Highly significant), *** (Very highly significant).

## Data Availability

The data presented in this study are available on request from the corresponding author.
